# Editorial: Community series in cell network in antitumor immunity of pediatric and adult solid tumors, volume II

**DOI:** 10.3389/fimmu.2025.1649643

**Published:** 2025-06-27

**Authors:** Ombretta Melaiu, Elin Bernson, Silvia Pesce

**Affiliations:** ^1^ Department of Clinical Sciences and Translational Medicine, University of Rome Tor Vergata, Rome, Italy; ^2^ Sahlgrenska Center for Cancer Research, University of Gothenburg, Gothenburg, Sweden; ^3^ Department of Obstetrics and Gynecology, Institute of Clinical Sciences, Sahlgrenska Academy, University of Gothenburg, Gothenburg, Sweden; ^4^ Department of Experimental Medicine University of Genoa, IRCCS Ospedale Policlinico San Martino, Genova, Italy

**Keywords:** tumor microenvironment (TME), tumor-infiltrating immune cells, biomarkers, tumor immunity, immunotherapy

This Research Topic has been curated to provide insights into the intricate interactions within the tumour microenvironment (TME) and their impact on cancer progression and treatment outcomes. The Research Topic features a variety of studies focused on impact that different immune and stromal cell types have in solid tumours, shedding light on emerging immunotherapeutic strategies and multi-omics approaches that enhance our understanding of this intricate field. This Research Topic specifically includes four original research articles, one case report, two brief research communications, and three comprehensive literature reviews.

Early cancer detection remains a cornerstone of improved oncology outcomes (1). Two studies both by Rebaudi et al. and Rebaudi et al., one on oral squamous cell carcinoma (OSCC) (Rebaudi et al.) and the other on oral leukoplakia (Rebaudi et al.), which is a precursor to potentially malignant oral disorders (PMOD), demonstrated the feasibility and reliability of non-invasive sampling using a cytobrush in conjunction with biomarker analysis. Using a customised ELISA immunoassay, the researchers could distinguish between malignant and healthy tissue by analysing selected biomarkers, such as EGFR, Ki67, p53, and immune checkpoint-related proteins (PD-L1, HLA-E, and B7-H6). These results corroborate histopathological gold standards and provide dynamic immunophenotypic data from living tissue.

Another key factor in gauging the severity of a tumour is pinpointing crucial genetic and immunohistochemical markers (2). Biatta et al. examined the concordance between *TP53* mutations and p53 immunohistochemical staining patterns in high-grade serous ovarian carcinoma (HGSOC). Null p53 expression, which corresponds to disruptive *TP53* mutations, were associated with significantly reduced overall survival. This offers clinicians an immunohistochemical surrogate marker over genetic analysis for this aggressive disease. Meanwhile, a review of neuroblastoma by D’Amico et al., explored the remarkable cellular plasticity that enables tumour cell transition between adrenergic (ADRN) and mesenchymal (MES) states. These transitions impact therapeutic resistance and immunogenicity, with MES cells emerging as favourable immunotherapy targets in scenarios where strategies mitigate interconversion and facilitate immune engagement.

Interestingly, a significant portion of this editorial’s work has focused on the importance of natural killer (NK) cells, which are pivotal players in the anti-tumour immune system (3). For instance, one of the most notable contributions in this Research Topic is the identification of PLAC1 as a novel ligand for several NK cell-activating receptors (NKARs), including NKG2D and NKp30. Romania et al. demonstrated through a high-throughput shRNA screen that PLAC1 expression enhances NK cell-mediated cytotoxicity. The overexpression of PLAC1 in a wide range of tumours, while remaining largely absent in normal tissues, makes it an ideal candidate for both biomarker development and immunotherapeutic targeting.

Yet, the TME often presents formidable immunosuppressive barriers (4). Karlsson et al., revealed Galectin-3 as a key immune modulator found in ascites and cyst fluid of patients with HGSOC. By priming neutrophils to produce reactive oxygen species (ROS), Galectin-3 indirectly impairs NK cell function, reinforcing the need for strategies that can neutralize suppressive signals within the TME.

Meanwhile, Mariotti et al. have improved our understanding of NK cell regulation by discovering that soluble PD-1 (sPD-1) enhances NK cell cytotoxicity. Produced endogenously by NK cells themselves, sPD-1 binds PD-L1 and suggests a novel autocrine or paracrine regulatory mechanism with potential therapeutic value. Animal models, too, are evolving to meet the demands of next-generation immunotherapy research (5). A comprehensive review by Parodi et al., maps the trajectory of NK cell studies from traditional murine systems to sophisticated humanized mouse models, reinforcing their continued importance in translational immunology.

In addition to NK cells, other components of the TME were also considered, including macrophages and neutrophils (6, 7). In this regard, a comprehensive review by Di Ceglie et al., on the roles of macrophages and neutrophils underscores their dual potential to either support or suppress tumour growth, depending on the local cytokine milieu and cell-cell interactions. As key modulators within the TME, their crosstalk with other immune cells, including NK cells, can influence the success of immunotherapies. Authors suggested that targeting these interactions holds promise as a novel means of reprogramming the immune landscape to favour anti-tumour responses.

Finally, in the case of pancreatic ductal adenocarcinoma (PDAC), a notoriously aggressive form of cancer (8), the dense desmoplastic stroma, which is driven by cancer-associated fibroblasts (CAFs), impedes the penetration of therapeutics and modulates immune responses. Using imaging mass cytometry, Erreni et al. profiled CAF subpopulations with unprecedented resolution and identified 19 phenotypically distinct clusters. Notably, specific CAF populations, such as CAFs 10 and 11, were enriched at the tumour-stroma interface and were associated with poorer prognoses. These fibroblasts co-localised with CD44+ macrophages and contributed to extracellular matrix remodelling, creating barriers to T cell infiltration. These findings highlight the functional heterogeneity of CAFs and their potential as therapeutic targets, particularly in efforts to re-engineer the tumour stroma to improve drug delivery and immune infiltration.

Together, these studies provide a roadmap for achieving a more systems-level understanding of cancer, from diagnosis through treatment. The convergence of non-invasive diagnostics, microenvironmental mapping, and molecular stratification paves the way for personalized interventions that not only target the tumour but reshape the ecosystem in which it thrives.

Whether through NK cell modulation, CAF profiling, or non-invasive biomarker discovery, the unifying theme is clear: precision oncology must be built on a foundation of deep biological insight, real-time monitoring, and therapeutic flexibility.
